# Health‐Related Quality of Life in Adult Patients With von Willebrand Disease From Germany: Results of the WIL‐QoL Study

**DOI:** 10.1111/hae.70073

**Published:** 2025-11-28

**Authors:** Sylvia von Mackensen, Carolin Moorthi, Ronald Fischer, Susan Halimeh, Christine Heller, Wolfgang Miesbach, Freimut H. Schilling, Cornelia Wermes, Guenter Auerswald, the WIL‐QoL study group, Karin Beutel, Georg Goldmann, Bettina‐Kemkes Matthes, Robert Klamroth, Ralf Knöfler, Christoph Male, Johannes Oldenburg, Ute Scholz

**Affiliations:** ^1^ Department of Medical Psychology University Medical Centre of Hamburg‐Eppendorf Hamburg Germany; ^2^ Coagulation Centre, Bremen Central Clinic GeNo Ltd., Parent‐Child‐Centre Prof. Hess Bremen Germany; ^3^ Haemophilia Centre University Hospital Giessen/Marburg Giessen Germany; ^4^ SRH Haemophilia Treatment Centre SRH Kurpfalz‐Hospital Heidelberg Heidelberg Germany; ^5^ Coagulation Centre Rhine‐Ruhr Duisburg Germany; ^6^ Department of Paediatrics, Paediatric Haemophilia Centre, Goethe University University Hospital Frankfurt Frankfurt/Main Germany; ^7^ Medical Clinic II, Institute of Transfusion Medicine, Goethe University University Hospital Frankfurt Frankfurt/Main Germany; ^8^ Children´s Hospital Olgahospital Stuttgart Germany; ^9^ Children's Hospital of Central Switzerland Lucerne Switzerland; ^10^ Department of Paediatric Haematology and Oncology Hanover Medical School Hanover Germany; ^11^ Haemophilia centres Hildesheim‐Hanover‐Osnabrück Hanover Germany; ^12^ Department of Paediatric Haematology and Oncology, University Hospital, Muenster, Germany, and Department of Paediatrics, Kinderklinik Muenchen Schwabing, Muenchen Klinik and Klinikum Rechts der Isar Technical University of Munich Munich Germany; ^13^ Institute of Experimental Haematology and Transfusion Medicine University Hospital Bonn Bonn Germany; ^14^ Haemophilia Centre University Hospital Giessen/Marburg Giessen Germany; ^15^ Clinic for Internal Medicine, Angiology, and Haemostaseology Vivantes Friedrichshain Hospital Berlin Germany; ^16^ Department for Haemostaseology, Clinic and Polyclinic for Paediatric and Adolescent Medicine University Hospital Carl Gustav Carus Dresden Germany; ^17^ Coagulation Unit, Department of Paediatrics and Adolescent Medicine Medical University of Vienna Vienna Austria; ^18^ Centre of Haemostasis MVZ Labor Leipzig Leipzig Germany

**Keywords:** bleeding score, disease‐specific questionnaire, health‐related quality of life, long‐term prophylaxis, VWD‐QoL, VWD type

## Abstract

**Introduction:**

Assessment of health‐related quality of life (HRQoL) is relatively new in von Willebrand disease (VWD). So far, generic questionnaires have mainly been used for HRQoL assessment in VWD.

**Aims:**

To assess generic and disease‐specific HRQoL in adult VWD patients and compare HRQoL with the general German population.

**Methods:**

Patients presenting with a personal or family history of bleeding and von Willebrand factor (VWF)‐specific laboratory parameters were enrolled in the WIL‐QoL study. HRQoL was assessed with generic (SF‐36) and disease‐specific (VWD‐QoL) questionnaires. Descriptive and inferential statistical procedures were applied based on a significance level at α ≤ 5%.

**Results:**

In the retrospective, multicentre WIL‐QoL study, HRQoL and clinical data in 120 adults with VWD (one adolescent completed the adult questionnaire) from 10 centres in Germany were collected. Compared to the corresponding age group in the general German population, female VWD patients had significantly worse HRQoL in all SF‐36 domains and male patients only in the ‘physical functioning’ domain. In the VWD‐QoL, highest impairments were seen in all VWD patients in the domains ‘other physicians’, ‘treatment’ and ‘sport & leisure’. VWD patients with a more significant disease burden, such as a bleeding score ≥ 9 (*p* < 0.0001), long‐term prophylaxis (*p* = 0.003), and VWD‐type 3 (*p* = 0.022), reported significantly worse HRQoL. No HRQoL differences were seen between male and female VWD patients.

**Conclusion:**

Female VWD patients showed significant impairments in their HRQoL compared to the age group‐related general population. Compared to SF‐36, the VWD‐QoL identified stronger significant HRQoL differences in most VWD subgroups, confirming the impact of bleeds.

## 1 Introduction

Quality of life (QoL) was first mentioned in connection with von Willebrand's Disease (VWD) in the clinical practice guidelines of the Association of Hemophilia Clinic Directors of Canada almost 30 years ago, suggesting that disease management should not only prevent disease and treatment‐associated morbidity and mortality but should also optimise QoL [1, 2].

In a recent systematic review of the epidemiology, burden of disease and management of VWD, the authors noted that VWD is a common disease, affecting 108.9 to 2200 per 100,000 in population‐based studies and 0.3 to 16.5 per 100,000 in referral‐based studies [2]. Compared to the general population, poorer health‐related quality of life (HRQoL) was reported in patients with VWD. Although all genders are equally affected by VWD, women tend to suffer more from VWD than male patients due to their menstrual bleeding [3, 4, 5, 6], especially those who suffer from heavy menstrual bleeding (HMB) [7].

HMB is not limited to VWD patients and is reported to constitute the most common symptom in women with bleeding disorders (WBD), manifesting as significant bleeding and pain [9, 10, 11]. HMB leads to limitations in conducting daily activities and changes in social functioning with an adverse effect on women's HRQoL [8, 9]. Due to the entrenched stigma and taboos, women and girls are often reluctant to discuss the problem of HMB with their families and do not seek medical advice. Since women often suffer in silence, only four in 10 women who perceive that they have excessive menstrual blood loss consult their doctor about it [12]. Studies have shown that WBD are more impaired in their HRQoL compared to healthy controls [10], especially women with HMB [11, 13, 14], women with VWD [11, 13, 14], and those who are currently menstruating [13, 14, 15].

Research on HRQoL is considered a relatively young area of medical research, which investigates wellbeing and function in physical, social and emotional domains [16]. Although VWD is the most common inherited bleeding disorder, the number of publications on HRQoL in VWD patients is relatively limited compared to the total number of publications in VWD [5, 17–21]. As for haemophilia, it has been demonstrated that bleeding patterns in VWD have an impact on patients’ HRQoL [18, 19, 20]; patients with a more severe VWD type had a significantly worse HRQoL than patients with a milder type [5, 19, 20]. The first disease‐specific HRQoL questionnaire for VWD patients (VWD‐QoL) was developed in Italy in 2007 [22] and subsequently linguistically validated for the French WiSH‐QoL study [23].

The aim of this part of the WIL‐QoL study was the assessment of HRQoL with the disease‐specific VWD‐QoL in adult VWD patients. Furthermore, HRQoL of the WIL‐QoL cohort was compared to the HRQoL of the general German population.

## 2 Material and Methods

### 2.1 Study Design and Patients

VWD patients were included in this retrospective, observational, cross‐sectional WIL‐QoL study, conducted at 13 centres in Germany and Austria, if they presented with an established diagnosis of VWD (personal or family history of bleeding symptoms and at least one of VWF‐specific laboratory parameters < 50% [such as VWF:Antigen, VWF:Ristocetin Cofactor, VWF:Collagen Binding] or a pathological multimer profile). A threshold of < 50% is in line with other publications [19, 24]. At the time of the study, the international guidelines on the diagnosis of von Willebrand disease were not available [25]. A convenience sample of patients visiting the respective haemophilia centres at their routine check‐ups between March 2011 and July 2013 was enrolled. Data were collected only for participating patients; therefore, no response rate is available. Patients were excluded if the inclusion criteria were not fulfilled or if pregnancy, acquired VWD, additional coagulopathies, concomitant diseases, or other diseases affecting HRQL were present, or if patients or their parents did not understand/sign the informed consent process and the study documents.

### 2.2 Study Procedures

To assess HRQoL, VWD patients self‐completed questionnaires during their routine visit at their treatment centre. HRQoL was assessed in three age groups: paediatric patients ≥ 4–7 years, paediatric patients 8–17 years and adult patients ≥ 18 years. Data in this manuscript will only report results for the adult WIL‐QoL cohort, involving 10 centres in Germany.

### 2.3 Patient‐Reported Outcomes (PRO) Measures

HRQoL was assessed with both a generic (Short‐form 36 [SF‐36] [26]) and a disease‐specific (Von Willebrand Disease‐specific Quality of Life Questionnaire [VWD‐QoL]) instrument. The SF‐36 consists of 36 items pertaining to eight domains: physical functioning (10 items), role physical (4 items), bodily pain (2 items), general health (5 items), vitality (4 items), social functioning (2 items), role emotional (3 items), and mental health (5 items). Transformed score values range from 0 to 100 with high scores implying a high HRQoL. Two component scores can be calculated: Physical Component Summary (PCS) and Mental Component Summary (MCS) [27]. The availability of norm data of the SF‐36 for Germany allows for a comparison of the WIL‐QoL cohort with the general population specific to gender and age [28].

Disease‐specific HRQoL was collected with the revised VWD‐QoL, which, for adult VWD patients, consists of 88 items covering 14 domains (no. of items): ‘treatment’ (9), ‘complaints’ (9), ‘physical’ (6), ‘feelings’ (13), ‘view’ (6), ‘family’ (5), ‘other’ (5), ‘sport & leisure’ (5), ‘work & school’ (4), ‘coping’ (7), ‘hospital’ (7), ‘other physicians’ (5), ‘future’ (4), and ‘relationship’ (3) [29]. Table 1 provides an overview of the underlying concepts and example items of each domain. Items were scored on a 5‐point Likert scale ranging from ‘never’ to ‘all the time’. To ensure that all responses were scored in the same direction, some items needed to be recoded reversely before calculating the domain scores. To be able to compare scale values of domains with different numbers of items, total and domain scores were transformed, ranging from 0 to 100, with lower scores indicating a better HRQoL [30]. The revised VWD‐QoL was validated for VWD patients in Germany (results presented in detail in the validation paper in this supplement [30]).

**TABLE 1:** Domains and examples of the VWD‐QoL questionnaire.

Domains	Underlying concept	Example item
Treatment	Treatment for VWD	*I had problems with the way in which my medication had to be administrated*
Complaints	Side effects caused by treatment for VWD (if applicable)	*Tingling after the infusion*
Physical	Physical health	*I had joint bleeds*
Feelings	Feelings about VWD	*I was worried because of my VWD*
View	Impact of VWD on self‐perception	*VWD made my life difficult*
Family	Relationships with the family in relation to the disease	*My family suffered because of my VWD*
Others	Relationships with other people in relation to the disease	*I felt different from other people because of my VWD*
Sport & leisure	Practice of sport and leisure	*Because of VWD I had to miss out on sports that I like*
Work & school	Impact of VWD on work and school aspects	*My everyday tasks at work/school were jeopardised by the VWD*
Coping	Attitude to deal with VWD	*I knew how I have to treat myself*
Hospital	Relationships with the hospital and hospital staff	*I felt dependent on the doctors because of the treatment of my VWD*
Other physicians	Relationships with physicians not specialised in VWD	*I have the feeling that other physicians not specialised in VWD underestimate the severity of VWD*
Future	Thoughts about the future life with VWD	*I worry that my condition could deteriorate*
Relationship	Impact of VWD on partnership	*I am unsure of my relationships with women/men because of my VWD*

### 2.4 Statistical Analyses

PROs were inserted into a database by Lampertius Daten‐Dienst Gesellschaft für Datenerfassung und Datenverarbeitung mbH, Munich, Germany, a company for data input and data processing. Data were cleaned by the lead author by checking for errors, implausible values, inconsistencies, multiple answers to a single question and missing information. Amendments were performed continuously in the database according to predefined rules.

Statistical analyses were performed using the SPSS program version 25 (Statistical Package for Social Science, IBM); differences between the VWD cohort and the German general population were analysed using the Statistics Kingdom program [31]. Data were analysed descriptively using contingency tables for categorical variables and sample statistics for continuous variables. Data are shown as mean ± standard deviation (M ± SD) for normally distributed data and median and ranges (minimum–maximum) for non‐parametric data distributions or counts and frequencies according to their distribution. Tests used for statistical significance had to be suitable for the data they analysed.

HRQoL differences between clinical subgroups were calculated using Student's T‐Test (ISTH bleeding score [BS] ≥ 9 vs. < 9 [based on median split]; long‐term prophylaxis: yes vs. no) or ANOVA (VWD type 1 vs. type 2 vs. type 3). Significance was set with *p* ≤ 0.05. Usually, tests were run two‐sided. We did not correct for multiple analyses (e.g., Bonferroni correction). In case of missing data exceeding 5%, we explicitly mentioned the exact numbers in the description (e.g., Figures) or footnote (e.g., Tables). If missing data did not exceed 5%, no relevant influence was assumed [32], and exact numbers were not reported.

## 3 Results

### 3.1 Socio‐Demographic Data and Family History

A total of 120 adult patients were enrolled in the HRQoL analyses from nine centres of the WIL‐QoL study. One 15‐year‐old adolescent patient had completed the adult VWD‐QoL questionnaire, and for one adult patient, only the HRQoL data were available and medical data were missing. Data from both patients were analysed together with the data of the adult group, which we consider an acceptable approach given that the threshold in other HRQoL questionnaires is 12 years, for example, in the SF‐36.

Table 2 lists socio‐demographic and family history data. Adult patients had an average age (M ± SD) of 38.7 ± 12.4 years, with women (37.8 ± 12.7 years) tending to be younger than men (43.5 ± 9.3 years; data not shown). Most patients reported that they have other family members suffering from VWD (87/116, 75%). However, only 5.3% (6/113) mentioned that they have a family member who is physically impaired/invalid due to VWD, and 6.1% reported a family member had died due to VWD (7/114).

**TABLE 2:** Socio‐demographic data of adult VWD patients (*n* = 120).

Socio‐demographic variable	*n* (%)
**Gender**	***n* = 120**
Male	20 (16.7)
Female	100 (83.3)
**Family status**	***n* = 118**
Single	31 (26.3)
Married	75 (63.6)
Divorced/separated	11 (9.3)
Widowed	1 (0.8)
**Living with a partner**	***n* = 120**
Yes	98 (81.7)
No	22 (18.3)
**Education** ^a^	***n* = 118**
Master's or equivalent	17 (14.4)
Bachelor's or equivalent	9 (7.6)
Upper secondary education	18 (15.3)
Lower secondary education	45 (38.1)
Primary education	27 (22.9)
No education	2 (1.7)
**Job**	***n* = 116**
Full‐time	43 (37.1)
Part‐time	31 (26.7)
Unemployed	10 (8.6)
Retired	12 (10.3)
Housewife/‐man	17 (14.7)
Student	3 (2.6)
**Missing days in past year**	***n* = 120**
Yes	91 (75.8)
Mean (SD)	8.28 (29.0)
Median (Min–Max)	0 (0–257)
**Other family members with VWD** ^b^	***n* = 116**
Yes	87 (75.0)
No	29 (25.0)
**Family members invalid due to VWD**	***n* = 113, 7 missing**
Yes	6 (5.3)
No	107 (94.7)
**Family members died due to VWD**	***n* = 114, 6 missing**
Yes	7 (6.1)
No	107 (93.9)

^a^ ISCED: International Standard Classification of Education 2011 [46].

^b^ Main other family members: brother (28.3%), mother (23.3%), father (16.7%), daughter (15.8%).

### 3.2 Self‐Reported Clinical Data – Medical History

Half of the VWD patients (49.6%, *n* = 59) stated that they had experienced bleeding in the preceding 4 weeks: mainly gum, nose and mouth bleeding, of which 11.9% reported joint bleeds (Table 3), correlating to only 5.9% of the total study cohort. Joint bleeds occurred across all types of VWD patients (type 1: *n* = 1 [1.6%]; type 2: *n* = 4 [9.3%]; type 3: *n* = 2 [15.4%]; data not shown).

**TABLE 3:** Self‐reported clinical data.

Medical history	*n* (%)
**Bleeding *in previous 4 weeks***	***n* = 119**
Yes	59 (49.6)
No	60 (50.4)
**Location of bleeds** ^a^	***n* = 59**
Gum	29 (49.2)
Nose	18 (30.5)
Mouth	15 (25.4)
Joints^b^	7 (11.9)
Faeces	5 (8.5)
Muscles	4 (6.8)
Throat	4 (6.8)
Abdomen	4 (6.8)
Urine	1 (1.7)
Others^c^	17 (29.8)
**Severity of bleeds *in previous 4 weeks***	***n* = 59**
Mild	31 (52.5)
Moderate	16 (27.1)
Severe	8 (13.6)
Extremely severe	4 (6.8)
**Tired *in previous 4 weeks***	***n* = 112, 8 missing**
Never	6 (5.4)
Seldom	17 (15.2)
Sometimes	35 (31.3)
Often	47 (42.0)
All the time	7 (6.3)
**VWD treatment** ^d^	***n* = 112, 8 missing**
No treatment	57 (50.9)
Intravenous	49 (43.8)
Oral	4 (3.6)
Oral and intravenous	2 (1.8)
**Perceived to receive the adequate treatment**	***n* = 101, 19 missing**
Yes	84 (83.2)
No	17 (16.8)
**Diagnosis**	***n* (%)**
**Feelings at diagnosis**	***n* = 113, 7 missing**
Cannot remember	43 (38.1)
Calmer/more balanced	20 (17.7)
Indifferent	22 (19.5)
Frustrated and scared about my future	28 (24.8)
**Restrictions after diagnosis**	***n* = 118**
Yes	46 (39.0)
No	72 (61.0)
**Type of restrictions after diagnosis** ^a^	***n* = 43**
Daily activities	29 (24.4)
Insurance issues	18 (15.1)
Job	12 (10.1)
Social contacts	5 (4.2)
Others^e^	15 (12.6)

^a^ Multiple answers possible.

^b^ Joint bleeds in previous 4 weeks [mild (*n* = 1), moderate (*n* = 4), severe (*n* = 2)].

^c^ mainly menstruations and traumatic bleeds.

^d^ Actual.

^e^ Limitations in sport (*n* = 6), pregnancy (*n* = 4), or medical procedures (*n* = 4).

Most patients (52.5%) reported mild bleeding, while 20.3% reported severe/extremely severe bleeding (*n* = 12). Forty‐eight per cent reported that they were ‘often’/’all the time’ tired in the previous 4 weeks. Half of the VWD patients took VWD medication (49%), mainly intravenously (44%; *n* = 49). Most patients (83.2%; *n* = 84) perceived getting adequate treatment.

### 3.3 Self‐Reported Clinical Data – Diagnosis

Age at diagnosis (M ± SD) of VWD (*n* = 115) was 24.6 ± 14.4 years with considerable variation (median 27, range: 0–64 years, data not shown). Mean time (± SD) between onset of symptoms and diagnosis was 13.8 ± 12.9 years (median 12, range 0–52 years, data not shown). Table 3 shows that most patients (*n* = 43) did not remember their feelings from the time of VWD diagnosis (38.1%), while one quarter recalled being frustrated at time of diagnosis (*n* = 28). Two‐fifths of the patients (39%; *n* = 46) experienced limitations after receiving the VWD diagnosis, mainly concerning daily activities (24.4%; *n* = 29) and insurance issues (15.1%; *n* = 18).

### 3.4 Self‐Reported Clinical Data – Menstruation

On average, female VWD patients (99/100) had their first menses at an age (M ± SD) of 12.7 ± 1.5 years (median 13 years, range: 10–16 years, data not shown). Most women (*n* = 71) were still experiencing menstruation (71%) with a duration (M ± SD) of 7.2 ± 2.9 days (median 7 days, range: 3–20 days; data not shown).

**FIGURE 1:**
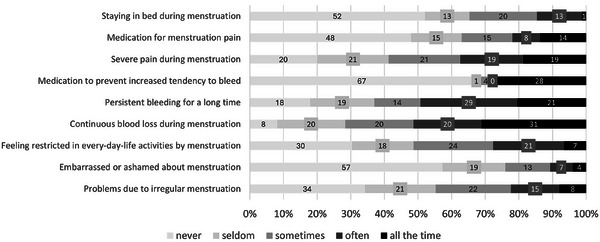
Aspects related to menstruation. Figure 1 shows the perception of female VWD patients related to their menstruation on a 5‐point Likert scale ranging from ‘never’ to ‘all the time’.

Concerning their menstruation, female patients mainly reported experiencing the following aspects ‘often’/‘all the time’: continuous blood loss (51.4%; *n* = 38), persistent bleeding for a long time (49.3%; *n* = 36), and severe pain (37.4%; *n* = 28, Figure 1).

### 3.5 HRQoL

#### 3.5.1 Generic HRQoL (SF‐36)

**FIGURE 2:**
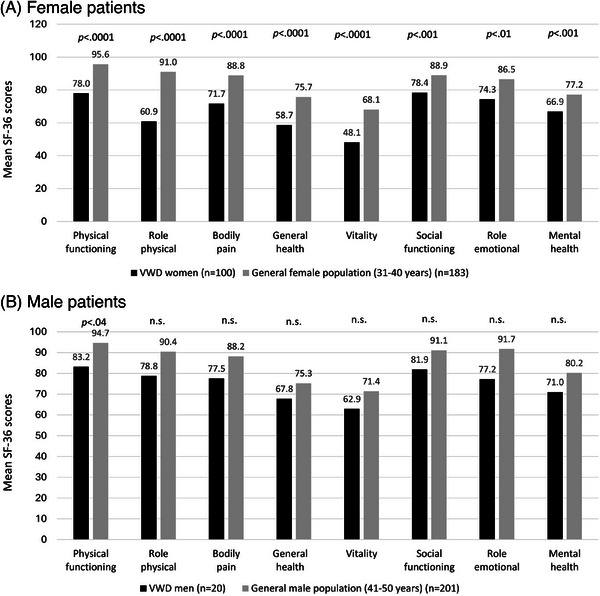
Comparison of generic HRQoL in adult VWD patients with the German general population (SF‐36) specified for gender and age. (A) Female VWD patients compared to women in the general population (31–40 years). (B) Male VWD patients compared to men in the general population (41–50 years). Figure 2 shows the mean HRQoL scores of adult VWD patients in the WIL‐QoL study compared to the German age‐ and gender‐related general population assessed with the generic SF‐36 questionnaire. Higher SF‐36 score values indicate better HRQoL. Statistically significant differences are indicated by *p* values.

VWD patients reported the highest impairments (M ± SD) in the generic SF‐36 domains ‘vitality’ (50.5 ± 23.3), ‘general health’ (60.2 ± 24.1) and ‘role physical’ (63.9 ± 42.2, data not shown). When stratifying by age and gender, female VWD patients experienced significantly worse HRQoL in all domains of the SF‐36 compared to the corresponding age group of women in the general German population (31–40 years of age) [28]. However, male VWD patients reported a significantly worse HRQoL in the SF‐36 domain ‘physical functioning’ compared to the corresponding age group of men in the general German population (41–50 years) (Figure 2A,B).

### 3.6 Disease‐Specific HRQoL (VWD‐QoL)

**FIGURE 3:**
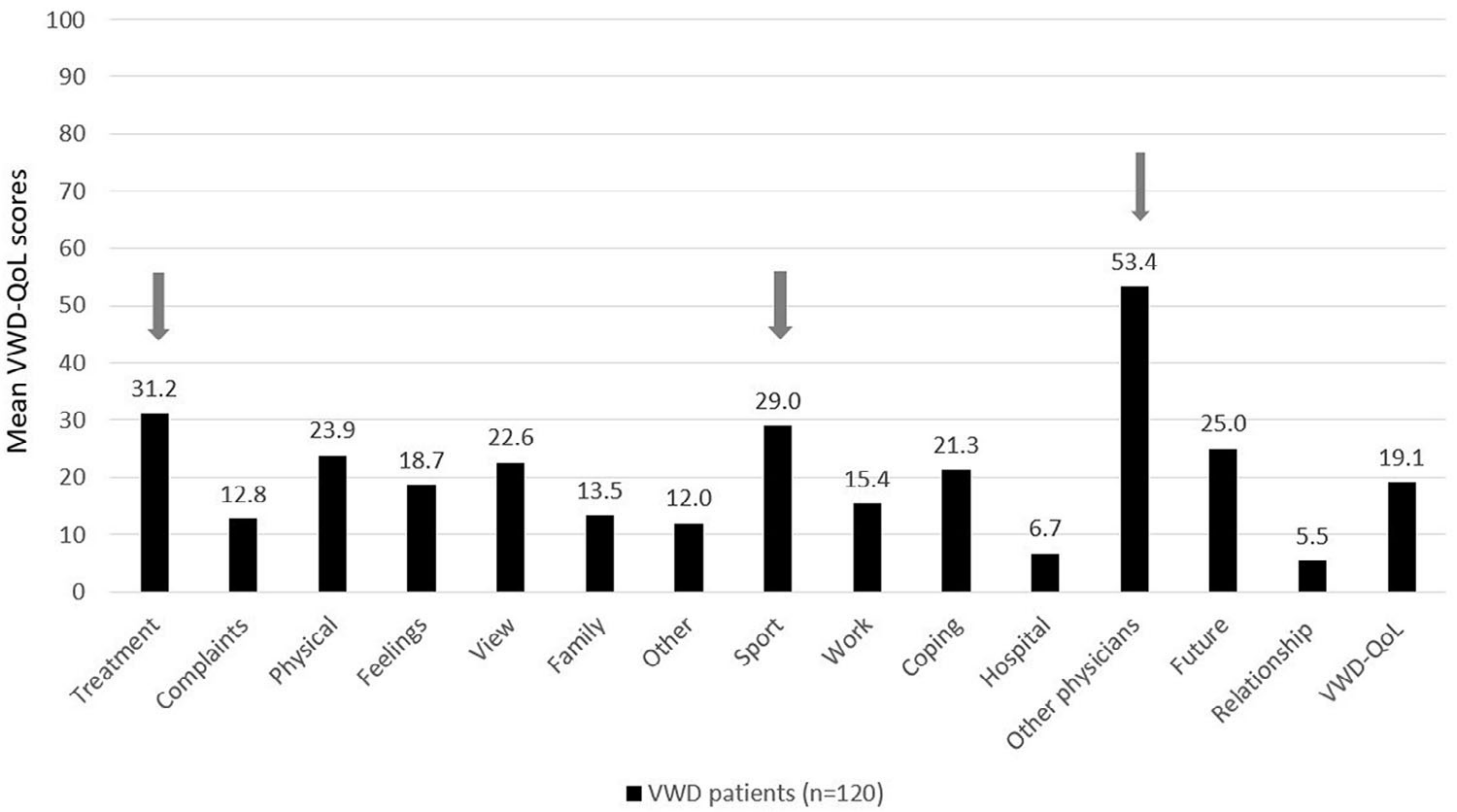
Disease‐specific HRQoL in adult VWD patients (VWD‐QoL). Figure 3 shows the mean HRQoL score of adult VWD patients (*n* = 120) assessed with the disease‐specific VWD‐QoL. Higher VWD‐QoL score values indicate higher impairments in HRQoL. Grey arrows highlight domains with the highest scores (worst HRQoL).

The disease‐specific HRQoL assessment demonstrated that VWD patients reported the highest impairments (M ± SD) in the VWD‐QoL domains ‘other physicians’ (53.39 ± 32.6), ‘treatment’ (31.19 ± 22.9) and ‘sport & leisure’ (29.04 ± 28.3) (Figure 3).

**FIGURE 4:**
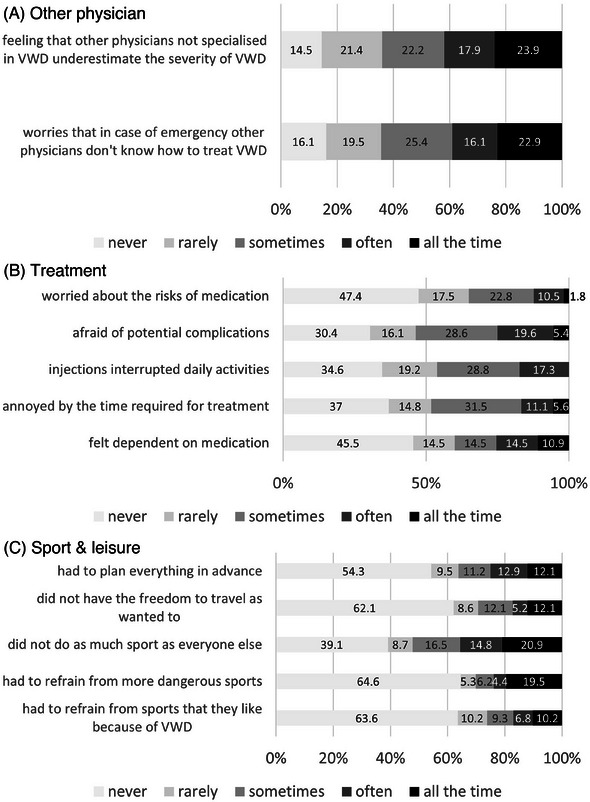
Domains with the highest HRQoL impairments (VWD‐QoL). (A) Other physicians. (B) Treatment. (C) Sport & leisure. Figure 4 shows the values of the individual items of the three VWD‐QoL dimensions with the strongest impairments (‘other physicians’, ‘treatment’ and ‘sport & leisure’).

Figure 4 displays the items of the three domains with the highest HRQoL impairments, showing the granularity of details affecting VWD patients in their everyday lives.

### 3.7 Differences in HRQoL Across Clinical Subgroups

**FIGURE 5:**
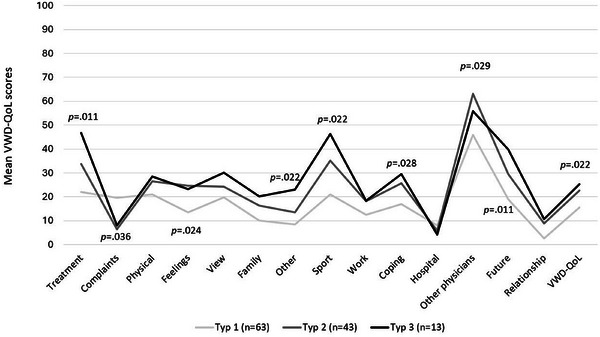
Differences in HRQoL regarding VWD type (VWD‐QoL). Figure 5 shows the mean VWD‐QoL scores across different VWD‐types such as type 1 (*n* = 63), type 2 (*n* = 43), and type 3 (*n* = 13). Higher VWD‐QoL score values indicate higher impairments in HRQoL. Statistically significant differences are indicated with *p* values.

HRQoL differences were analysed for clinical subgroups. Significant HRQoL differences were found for the VWD‐QoL results across different *types of VWD*; however, no significant differences were found with the SF‐36. Type 3 patients (*n* = 13) had the worst HRQoL in most VWD‐QoL domains compared to type 2 (*n* = 43) and type 1 (*n* = 63) patients (Figure 5).

**FIGURE 6:**
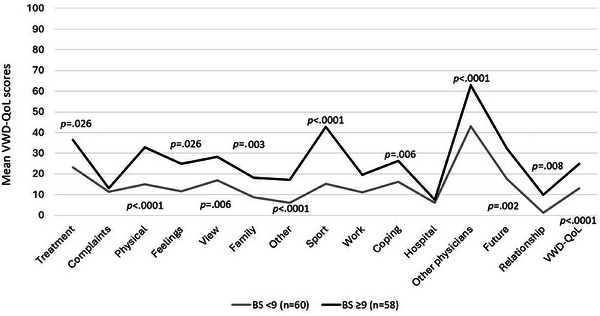
Differences in HRQoL regarding ISTH bleeding score (BS) in the last 12 months (VWD‐QoL). Figure 6 shows the mean VWD‐QoL scores for patients with an ISTH bleeding score (BS) ≥ 9 (*n* = 58) compared to those with a BS < 9 (*n* = 60). Higher VWD‐QoL score values indicate higher impairments in HRQoL. Statistically significant differences are indicated with *p* values.

VWD patients with an *ISTH BS* ≥ 9 (*n* = 58) reported a significantly worse HRQoL (M ± SD) compared to patients with a BS < 9 (*n* = 60) in the SF‐36 (PCS: 43.1 ± 12.4 vs. 51.62 ± 8.0; *p* < 0.0001) and in nearly all VWD‐QoL domains (*p* < 0.0001; Figure 6). Patients who reported that they had *bleeds in the preceding 4 weeks* (*n* = 59) had significantly worse HRQoL (M ± SD) in both the SF‐36 (PCS: 44.15 ± 12.3 vs. 50.43 ± 9.23; *p* = 0.004) and VWD‐QoL (23.86 ± 15.5 vs. 14.07 ± 13.9; *p* < 0.0001) compared to patients without bleeds (*n* = 60; data not shown). This became even more apparent in patients with *joint bleeds in the preceding 4 weeks* (*n* = 7), with HRQoL values in the SF‐36 (PCS: 29.0 ± 10.5 vs. 48.37 ± 10.4; *p* < 0.001) and VWD‐QoL (42.26 ± 13.9 vs. 17.67 ± 14.5; *p* < 0.0001) compared to patients without joint bleeds (*n* = 113) (data not shown).

**FIGURE 7:**
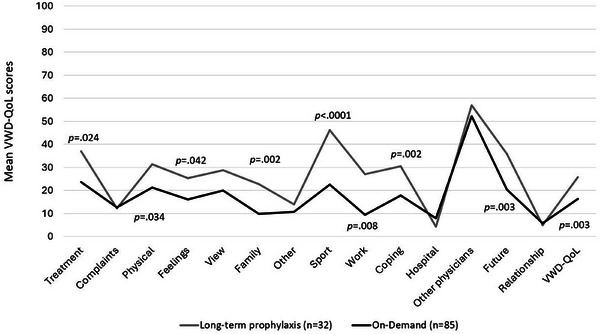
Differences in HRQoL regarding treatment (VWD‐QoL). Figure 7 shows the mean VWD‐QoL scores for patients receiving long‐term prophylaxis (*n* = 32) compared to those receiving on‐demand treatment (*n* = 85). Higher VWD‐QoL score values indicate higher impairments in HRQoL. Statistically significant differences are indicated with *p* values.

Patients receiving *long‐term prophylaxis* (*n* = 32; defined as regular prophylaxis including prophylaxis during HMB [33]) showed a significantly worse HRQoL compared to patients receiving on‐demand treatment (*n* = 85) in the SF‐36 (PCS: 41.55 ± 11.9 vs. 49.22 ± 10.6; *p* = 0.003) and in almost all VWD‐QoL domains (*p* < 0.0001, Figure 7).

No significant differences in HRQoL were seen in the VWD‐QoL for *gender*, although women still experiencing menstruation who self‐reported HMB (HMB‐PRO; *p* = 0.004) and impaired everyday life due to menstruation (*p* < 0.001) had a significantly worse HRQoL compared to women who did not report these issues (data presented in detail in the paper on women's issues in this supplement [34]).

## 4 Discussion

In the WIL‐QoL study, significant HRQoL impairments in the generic SF‐36 were shown between female VWD patients compared to women in the age‐related general German population; VWD men reported only worse HRQoL in the domain ‘physical functioning’ compared to the age‐related general male population. Other studies assessing HRQoL in VWD patients also revealed differences in SF‐36 domains compared to the general population, although differences in the domains varied across these studies [19, 20, 21]. Similar results using other generic instruments (such as the Health Utility Index Mark 3, EQ VAS) have also been described in the literature [35].

In the WIL‐QoL study, a subgroup analysis between VWD patients with a BS < 9 vs. BS ≥ 9 demonstrated a significant difference in the PCS of the SF‐36 and almost all domains of the VWD‐QoL. Similar findings were shown by de Wee et al., who compared VWD patients with a BS > 17 to patients with a BS < 7 [20]; patients with a high BS reported significantly lower HRQoL in all domains except the domain ‘mental health’ and the MCS (univariate analysis). Xu et al. also looked at the impact of BS and iron status on the HRQoL (SF‐36) of VWD patients [19]; after adjusting for age, sex, socioeconomic status, and rurality, they found a lower PCS trend with increasing BS and a lower MCS trend with iron deficiency status (*p* = 0.07 and *p* = 0.08, respectively).

Although in the WIL‐QoL study only seven patients reported joint bleeds, significant HRQoL differences were found in almost all domains of the SF‐36 and the VWD‐QoL. Van Galen et al. found that 23% of the 804 moderate and severe VWD patients (age range 0–85 years) in the Willebrand in the Netherlands (WiN) study experienced joint bleeds, which resulted in reduced HRQoL (SF‐36) [18]. As noted in a recent review on HRQoL in VWD, joint bleeding in severe VWD is more common than anticipated [36, 37] and, although expected to be more likely to occur in patients with severe haemophilia [36], it can be as debilitating [38].

While VWD type 3 patients in the WIL‐QoL study reported a significantly worse HRQoL in several domains of the disease‐specific VWD‐QoL compared to type 2 or 1 patients, this difference was not seen in the generic SF‐36, in contrast to findings in the paediatric Dutch WiN study [17] or the Canadian study [5] in patients ≥ 13 years, where a significant difference in the SF‐36 and Health Utilities Index Mark 3 (HUI3), respectively, was found between VWD types.

No differences between men and women with VWD were seen in the WIL‐QoL study, in contrast to the findings in the literature where VWD females have a greater morbidity burden than males with VWD [5, 7, 39]. Nevertheless, significant HRQoL differences were found in women in the WIL‐QoL cohort still experiencing menstruation, who reported impairments related to their menstruation such as impaired everyday life, self‐reported HMB (HMB‐PRO), and pain during menstruation compared to women who did not report these impairments (data published elsewhere in this supplement [34]), which mirrors the findings from other authors [5, 7, 9–12, 20, 21, 39]. This underlines the importance of improving HRQoL as an important goal in the treatment of HMB, which is in accordance with the National Institute for Health and Care Excellence guideline [40].

Compared to a generic questionnaire, the assessment of HRQoL in the WIL‐QoL study with a disease‐specific questionnaire (VWD‐QoL) emphasises its benefits to better detect HRQoL differences in patient subgroups. Interestingly, 42% of patients reported in the VWD‐QoL the feeling that physicians not specialised in VWD underestimate the severity of the disease (‘often’/‘all the time’), while 39% worried that, in case of emergency, other physicians would not know how to treat VWD.

Moreover, the VWD‐QoL could facilitate longitudinal assessment of patients to evaluate the impact of new treatment regimens on VWD patients’ HRQoL [20]. The only other study that uses the disease‐specific VWD‐QoL is the French Willebrand Study Health‐related Quality of Life (WiSH‐QoL) [23, 39]. A recent publication of this group in adult VWD patients (male and female) showed a tendency in all dimensions of the VWD‐QoL to be better in men except for ‘sport & leisure’; however, only ‘physical health’ appeared significantly different at baseline [39]. Moreover, they found that VWD type 3 patients were more affected compared to type 1 and type 2 patients, especially in the VWD‐QoL domains ‘physical health’, ‘feeling’, ‘family’ and ‘other persons’.

Why patients receiving long‐term prophylaxis presented with a reduced HRQoL in the VWD‐QoL remains to be investigated further. However, one explanation could be more severe VWD and inconsistencies in the regimen for patients receiving prophylaxis. This could be similar to treatment in the 2000s, when prophylaxis was only given to adult haemophilia patients on a case‐by‐case basis due to limited resources for haemophilia treatment and uncertainty as to whether it would stop or slow down the deterioration of haemophilic arthropathy in patients with joint damage [41, 42].

The WIL‐QoL study has some limitations. Patients’ answers were not always consistent; sometimes a recall bias occurred since patients could not correctly remember situations that happened in the past. Moreover, patient‐ and physician‐reported clinical data were not always coherent, which might be due to lacking accuracy of patient‐reported data, potentially influenced by their health literacy. In addition, patient reports and medical records each have the potential to over‐report or under‐report medical conditions [43]. It could also be considered critical that there was bias in patient enrolment, which is related to the convenience sampling method used to select the patient cohort. Although the prevalence of VWD between men and women is equal [44], only 1/5 of the adult patients enrolled in the WIL‐QoL study were men. This is not surprising considering that normally two‐thirds of VWD patients seen at haemophilia treatment centres are female, as they are more likely to experience symptoms of VWD [44]. However, whether the data for male VWD patients are representative remains questionable. Another limitation is related to the fact that the data are more than 10 years old. Prophylactic treatment has evolved and become more individualised in recent years, but, currently, there are no more recent published data available in Germany.

Thanks to the additional HRQoL assessment with the disease‐specific VWD‐QoL, a deeper insight into specific VWD‐related aspects could be gained than is possible with generic instruments. The disease‐specific HRQoL assessment reveals important health aspects, especially in VWD patient subgroups, which are potentially not yet receiving high priority.

## 5 Conclusion

HRQoL in female VWD patients was worse compared to the age‐related female general population, affecting both physical and mental domains. In the VWD‐QoL, significant HRQoL differences were also found in clinical subgroups such as VWD type, patients with an ISTH BS ≥ 9, patients with bleeds in the last 4 weeks (including joint bleeds) and patients receiving long‐term prophylaxis. Compared to the SF‐36, the VWD‐QoL identified greater significant HRQoL differences in almost all respective clinical subgroups, except for the ISTH BS or VWD type (for the latter, no significant differences were found in the SF‐36), pointing towards the superiority of disease‐specific HRQoL instruments compared to less‐sensitive generic instruments.

Therefore, the HRQoL assessment with a VWD‐specific questionnaire allows a deeper insight into how the specific disease pattern impacts patients’ HRQoL within subgroups of patients, extending the current scope of generic HRQoL instruments. The disease‐specific VWD‐QoL is an important HRQoL instrument, allowing the identification of VWD patients with the highest burden of disease and so fostering early, prospective and specific treatment according to patients’ needs in order to reduce future complications. More studies are needed in this important field of medicine.

## Author Contributions

Sylvia von Mackensen and Guenter Auerswald designed the study, Sylvia von Mackensen developed the VWD‐specific VWD‐QoL questionnaires, and Carolin Moorthi and Guenter Auerswald developed the *ad‐hoc* survey questionnaire to assess clinical data.

Susan Halimeh, Cornelia Wermes, Carolin Moorthi, Ronald Fischer, Christine Heller, Wolfgang Miesbach, Freimut H. Schilling, Guenter Auerswald, and the WIL‐QoL study collaborators recruited patients and reviewed answer sheets. All authors critically discussed the statistical data analyses. Sylvia von Mackensen drafted the initial manuscript. All authors critically revised and approved the final version of the document.

## Funding

We thank CSL Behring, Hattersheim, Germany, for providing financial support for the WIL‐QoL study and for the development of this supplement.

## Ethics Statement

We conducted this study following the International Conference on Harmonisation Good Clinical Practice guidelines. All centres submitted the study documents to the Institutional Review Board (IRB), and the study started once the responsible IRB had approved the study. All patients or their legal representatives signed written informed consent before the study start (see Moorthi et al. [45]).

## Conflicts of Interest

Sylvia von Mackensen received research funding from CSL Behring, Germany, to conduct the WIL‐QoL study; she also received research funding and speaker's honorarium from Grifols and LFB. Carolin Moorthi received a grant from CSL Behring, Germany, for scientific research. Ronald Fischer received research funding and personal fees from Bayer, Biotest, CSL Behring, Takeda/Shire, Novo Nordisk, Octapharma, Pfizer, Roche, Sobi. Susan Halimeh received research funding and personal fees from Bayer, Baxalta/Shire/Takeda, Biotest, CSL Behring, Novo Nordisk, Octapharma, Pfizer, Roche, Sobi. Christine Heller received research funding and personal fees from Biotest, Roche, CSL Behring, Pfizer, Sobi, Takeda/Shire, Bayer. Wolfgang Miesbach received research funding and personal fees from Bayer, Biomarin, Biotest, CSL Behring, Chugai, Freeline, LFB, Novo Nordisk, Octapharma, Pfizer, Roche, Sanofi, Takeda/Shire, uniQure. Freimut H. Schilling received research funding, speaker's honorarium, consultation fees and travel grants from Bayer, CSL Behring, Novo Nordisk, Pfizer, Roche, Sobi, Takeda/Shire. Cornelia Wermes received personal fees from Bayer, Biotest, CSL Behring, LFB, Novo Nordisk, Pfizer, Sobi, Takeda. Guenter Auerswald has received research funding from CSL Behring for the conduct of the WIL‐QoL study.

## Data Availability

The data that support the findings of this study are available on request from the WIL‐QoL core team (please mail to: s.mackensen@uke.de). The data are not publicly available due to privacy or ethical restrictions.
